# Preclinical efficacy and clinical safety of clinical‐grade nebulized allogenic adipose mesenchymal stromal cells‐derived extracellular vesicles

**DOI:** 10.1002/jev2.12134

**Published:** 2021-08-14

**Authors:** Meng‐meng Shi, Qing‐yuan Yang, Antoine Monsel, Jia‐yang Yan, Cheng‐xiang Dai, Jing‐ya Zhao, Guo‐chao Shi, Min Zhou, Xue‐mei Zhu, Su‐ke Li, Ping Li, Jing Wang, Meng Li, Ji‐gang Lei, Dong Xu, Ying‐gang Zhu, Jie‐ming Qu

**Affiliations:** ^1^ Department of Pulmonary and Critical Care Medicine Rui‐jin Hospital Shanghai Jiao‐tong University School of Medicine Shanghai China; ^2^ Institute of Respiratory Disease Shanghai Jiao‐tong University School of Medicine Shanghai China; ^3^ Key Laboratory of Emergency Prevention Diagnosis and Treatment of Respiratory Infectious Diseases Shanghai China; ^4^ Multidisciplinary Intensive Care Unit Department of Anaesthesiology and Critical Care La Pitié‐Salpêtrière Hospital Assistance Publique‐Hôpitaux de Paris (APHP) Sorbonne University France; ^5^ INSERM Sorbonne Université UMR S 959, Immunology‐Immunopathology‐ Immunotherapy (I3); F‐75005 Paris France; ^6^ Biotherapy (CIC‐BTi) and Inflammation‐Immunopathology‐Biotherapy Department (DHU i2B) Hôpital Pitié‐Salpêtrière AP‐HP, F‐75651 Paris France; ^7^ Cellular Biomedicine Group Inc. (CBMG) Shanghai China; ^8^ Daxing Research Institute University of Science and Technology Beijing Beijing China; ^9^ Department of Pulmonary and Critical Care Medicine Hua‐dong Hospital Fudan University Shanghai China

**Keywords:** extracellular vesicles, healthy volunteers, lung injury, mesenchymal stromal cells, nebulization

## Abstract

Mesenchymal stromal cell‐derived extracellular vesicles (MSC‐EVs) turn out to be a promising source of cell‐free therapy. Here, we investigated the biodistribution and effect of nebulized human adipose‐derived MSC‐EVs (haMSC‐EVs) in the preclinical lung injury model and explored the safety of nebulized haMSC‐EVs in healthy volunteers. DiR‐labelled haMSC‐EVs were used to explore the distribution of nebulized haMSC‐EVs in the murine model. *Pseudomonas aeruginosa*‐induced murine lung injury model was established, and survival rate, as well as WBC counts, histology, IL‐6, TNF‐α and IL‐10 levels in bronchoalveolar lavage fluid (BALF) were measured to explore the optimal therapeutic dose of haMSC‐EVs through the nebulized route. Twenty‐four healthy volunteers were involved and received the haMSC‐EVs once, ranging from 2 × 10^8^ particles to 16 × 10^8^ particles (**MEXVT study, NCT04313647**). Nebulizing haMSC‐EVs improved survival rate to 80% at 96 h in *P. aeruginosa*‐induced murine lung injury model by decreasing lung inflammation and histological severity. All volunteers tolerated the haMSC‐EVs nebulization well, and no serious adverse events were observed from starting nebulization to the 7th day after nebulization. These findings suggest that nebulized haMSC‐EVs could be a promising therapeutic strategy, offering preliminary evidence to promote the future clinical applications of nebulized haMSC‐EVs in lung injury diseases.

## 1 INTRODUCTION

Acute respiratory distress syndrome (ARDS) is characterized by diffuse alveolar damage, accounting for 10% of intensive care unit (ICU) admissions, and is present in 25% of mechanically ventilated patients (Fan et al., 2018; Thompson et al., 2017). A variety of clinical disorders can cause ARDS, such as severe trauma, pneumonia, sepsis and pancreatitis (Matthay et al., 2019). Despite management mainly based on supportive measures, such as protective mechanical ventilation, neuromuscular blocking agents or prone positioning, worldwide ARDS mortality remains high, around 30%.

Mesenchymal Stromal cells‐derived extracellular vesicles (EVs) were EVs secreted by MSCs at rest or under distinct stimulations (Ratajczak et al., 2006), such as hypoxic stress, irradiation, oxidative damage, or serum starvation. EVs are regarded as significant signalling mediators for intercellular communication, partially due to their ability to transport particular proteins, mRNA, and microRNA (Camussi et al., 2010). Multiple preclinical studies supported MSC‐EVs to be a potential therapeutic strategy for ALI caused by different types of non‐infectious factors, such as LPS (Kaspi et al., 2021), smoke inhalation (Xu et al., 2020), burn (Liu et al., 2019) and trauma (Li et al., 2019). Besides, there is also a large body of evidence demonstrating that lung inflammation resulting from tracheal inoculation of live *Escherichia coli* (Curley et al., 2017; Devaney et al., 2015; Gupta et al., 2012; Kim et al., 2011; Lee et al., 2013; Monsel et al., 2015; Park et al., 2019; Sung et al., 2016) and *Pseudomonas aeruginosa* (PA) (Mao et al., 2015) is significantly reduced by MSCs (Curley et al., 2017; Devaney et al., 2015; Gupta et al., 2012; Kim et al., 2011; Lee et al., 2013; Mao et al., 2015; Sung et al., 2016) and MSC‐EVs (Monsel et al., 2015; Park et al., 2019). The macrophage phagocytosis (Devaney et al., 2015; Lee et al., 2013; Mao et al., 2015; Monsel et al., 2015; Park et al., 2019; Sung et al., 2016), bacterial killing (Curley et al., 2017; Devaney et al., 2015; Gupta et al., 2012; Kim et al., 2011; Lee et al., 2013; Mao et al., 2015; Monsel et al., 2015; Park et al., 2019; Sung et al., 2016) and outcome (Curley et al., 2017; Devaney et al., 2015; Gupta et al., 2012; Kim et al., 2011; Mao et al., 2015; Monsel et al., 2015) are improved, suggesting similar benefits of MSC‐EVs administration as MSCs for the treatment of inoculation pneumonia.

MSC‐EVs own similar therapeutic properties to MSCs in ALI models, with more accessibility to be prepared, stored and delivered to the bedside while avoiding certain cell‐therapy limitations, such as the risk of pulmonary embolism and tumour formation. In recent years, MSC‐EVs have attracted much attention because of their potential role as a biomarker in pathogenesis and as a therapeutic agent for various diseases. Several clinical studies suggested that the administration of MSCs (up to a dose of 10^7^ cells/kg of body weight) could be a possible treatment for ARDS (Matthay et al., 2019; Wilson et al., 2015; Zheng et al., 2014). Several clinical trials manifested that intravenous infusion of MSCs and MSC‐EVs was safe and effective for severe lung injury caused by SARS‐CoV‐2 (Meng et al., 2020; Shu et al., 2020).

So far, most published clinical trials about MSCs and MSC‐EVs focusing on ARDS were administered intravenously. In these studies, although the safety profile of MSCs and MSC‐EVs treatment was suggested to be correct, the nonsignificant therapeutic effect might lie in the route of administration. The nebulized route constitutes a particularly interesting route of administration in the context of lung damage, given its excellent performance in terms of the bioavailability of the drug delivered to the targeted pulmonary site. This pilot study aims to investigate the distribution and optimum dose of nebulized human adipose‐derived MSC‐Extracellular vesicles (haMSC‐EVs) in a preclinical murine model, as well as to explore the safety of nebulized haMSC‐EVs in healthy volunteers. We aim to offer some evidence to promote the future clinical applications of nebulized haMSC‐EVs in ARDS.

## 2 MATERIALS AND METHODS

### 2.1 Primary cells and cell lines cultures

Clinical‐grade human adipose‐derived MSCs‐Extracellular Vesicles (haMSC‐EVs) were obtained from Cellular Biomedicine Group, Inc. (CBMG, Shanghai, China, https://www.cellbiomedgroup.com). The detailed information of haMSCs was shown in Supplementary methods. NCTC clone 929 cells (L cell, L‐929, a derivative of Strain L) (Catalog #GNM28, http://www.cellbank.org.cn/detail_1.asp?id = 352&serial = GNM28) were obtained from the cell bank of the Chinese Academy of Sciences. The L‐929 cells were cultured in RPMI1640 (Gibco, Thermo Fisher Scientific, Waltham, MA, http://www. thermofisher.com). Isolation and characterization of L‐929 EVs were performed according to the protocols previously described (Shi et al., 2021).

### 2.2 Mice

C57BL/6 and BALB/c male mice (7–8 weeks) were obtained from Shanghai Laboratory Animal Co. Ltd., China. Animals were kept in a specific pathogen‐free facility of the Research Centre for Experimental Medicine of Rui‐jin Hospital, Shanghai Jiao‐tong University School of Medicine, China. All animal procedures were approved by the Rui‐jin hospital Animal Ethics Committee.

### 2.3 Identification and preparation of clinical strain

The *P. aeruginosa* strain, obtained from Rui‐jin Hospital, Shanghai Jiao‐tong University School of Medicine, was isolated from the sputum of a hospitalized patient with pneumonia. Genome analysis was also conducted by next‐generation sequencing (NGS) (Shanghai Personal Biotechnology Co. Ltd. https://www.personalbio.cn), and the strain was demonstrated to match with serotype of O11 (Accession number 104721.1) by BLAST (Basic Local Alignment Search Tool, https://blast.ncbi.nlm.nih.gov/Blast.cgi?PROGRAM = blastn&PAGE_TYPE = BlastSearch&LINK_LOC = blasthome).

### 2.4 Isolation and characterization of haMSC‐EVs

**FIGURE 1:**
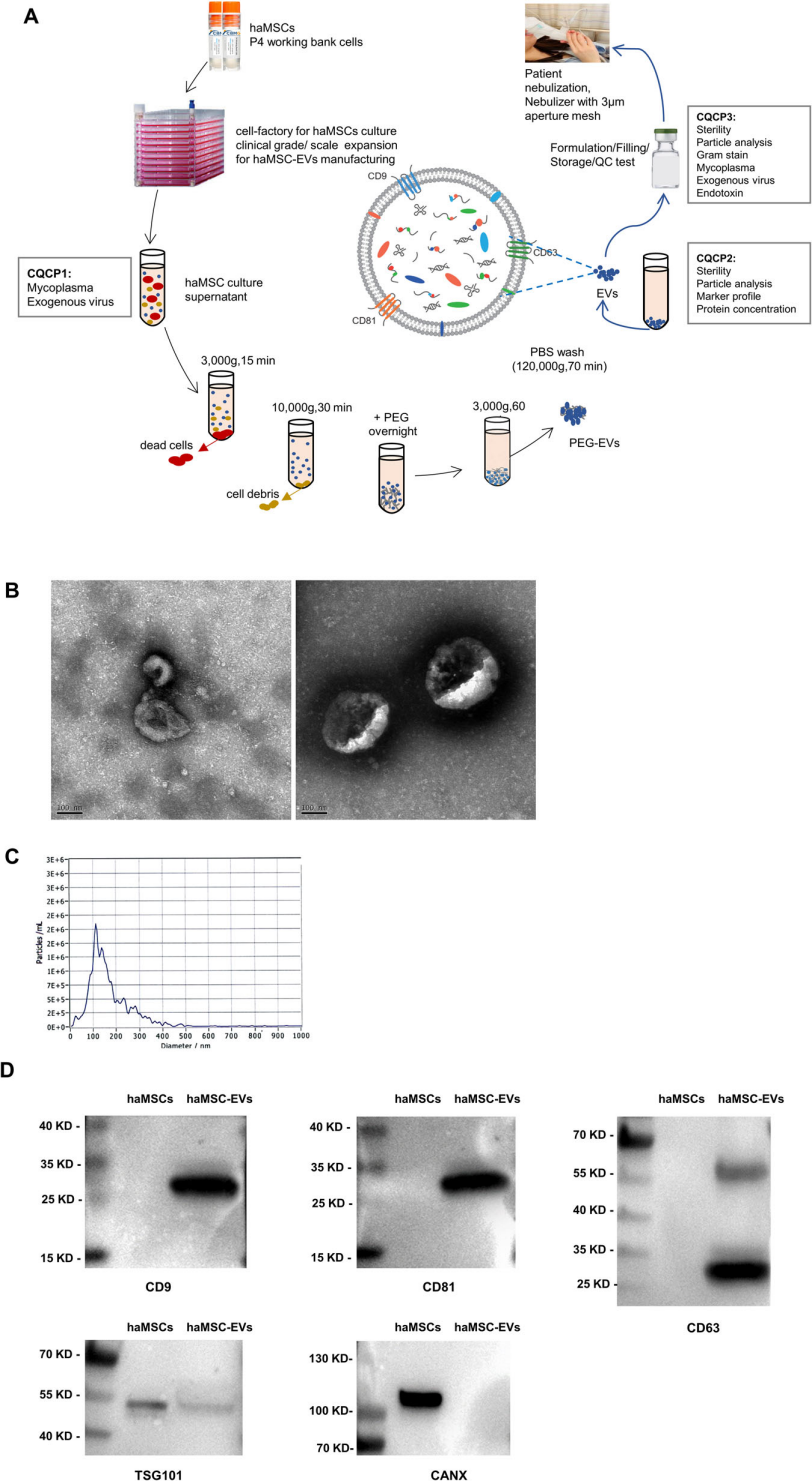
The manufacture and characterization of haMSC‐EVs. (a) haMSC‐EVs manufacture. (b) Representative electron microscopic photograph of haMSC‐EVs, scale bar = 100 nm. (c) The concentration and size distribution of haMSC‐EVs were determined by NTA. (d) Representative western blots showing the expression of EV markers, including CD9, CD81, CD63, TSG101, and CANX. haMSC‐EVs: human adipose‐derived MSC‐Extracellular vesicles; NTA: Nanoparticle Tracking Analysis

The detailed process of manufacture and quality control of haMSC‐EVs were presented in Figure 1a. The haMSCs were cultured to fourth passages and were seeded at 1–1.5 × 10^4^/cm^2^ density. When cells reached 90% confluence, the complete medium (α‐MEM containing human platelet lysate) was changed to condition medium (Condition medium was defined as EV‐free medium). The complete medium was centrifuged at 120,000 g for 6 h to deplete EVs. And the supernatant was taken as the EV‐free medium. We used condition medium to incubate cells for EV harvest and did not use serum starvation). After incubation for 48 h, the supernatant was depleted of cell debris by differential centrifuge, incubated with 12% polyethylene glycol (PEG) for 24 h, and was centrifuged at 3000 g for 1 h at 4°C. The pellet was re‐suspended by phosphate buffer saline (PBS) and centrifuged at 120,000 g for 70 min to deplete free protein and impurities. The haMSC‐EVs were finally solved with saline. We have established a system to make sure Good Manufacturing Practice (GMP) criteria were met at each manufacturing step, including facility certification, personnel training, material control, environmental monitoring, and relative document record management. The Critical Quality Control Points (CQCP) at the critical process step were labelled in Figure 1a. The tests at each CQCP were listed in Table 1. Cell and extracellular vesicles lysates were prepared with NP40 lysis buffer (Thermo Scientific), and the protein concentration was determined by BCA assay (Beyotime). Immunoblot assays were conducted according to the standard protocol with MV lysates (10—15 μg proteins per lane), using the following antibodies, primary antibodies: CD9 (Catalogue No.AHS0902), CD81 (Catalogue No.10630D), CD63 (Catalogue No.10628D) (Thermo); TSG101 (Catalogue No.ab125011) (Abcam); CANX (Catalogue No.A15631) (Abclonal); secondary antibodies: Anti‐Mouse IgG (Whole Molecule) Peroxidas E Cconjugat (Catalogue No.A9044‐2ML) (Sigma), Anti‐rabbit IgG, HRP‐linked Antibody (Catalogue No.7074S) (Cell Signalling). The signal was developed by ECL (Merk). Measurements of particle size distribution and concentration were performed with ZetaView PMX 120 (Particle Metrix, Meerbusch, Germany) based on a Nanoparticle Tracking Analysis (NTA). The ZetaView Nanoparticle Tracking Analyzer was calibrated with polystyrene microspheres. 10 μl samples were diluted with 10 ml PBS for detection and were analysed by ZetaView. A 20–40 μl solution of EVs was placed on a carbon‐coated copper grid and post‐negatively stained with 2% uranyl acetate for 10 min. The sample was then dried for 2 min. The grid was observed and photographed under a transmission electron microscopea

**TABLE 1:** Tests at each critical quality control point

Parameter	Release criteria	Method
CQCP1:Cell culture supernatant		
Mycoplasma	Negative	qPCR
Exogenous virus	Negative	According to ChP
CQCP2: Drug substance		
Sterility	Negative	BacT/ALERT
Mycoplasma	Negative	qPCR
Exogenous virus	Negative	According to ChP
Particle analysis	50‐400 nm particles > 80%	NTA
Marker profile	CD9^+^,CD63^+^,CD81^+^, TSG101^+^,CANX^−^	Western blot
Protein concentration	–	BCA
CQCP3: Preparations		
Sterility	Negative	BacT/ALERT
Gram's staining	Negative	Microscope
Endotoxin	<100 EU/ml	Limulus assay
Mycoplasma	Negative	qPCR
Exogenous virus	Negative	According to ChP
Particle analysis	Labelled amount ±20%	NTA

### 2.5 Experimental murine lung injury model with nebulized haMSC‐EVs

**FIGURE 2:**
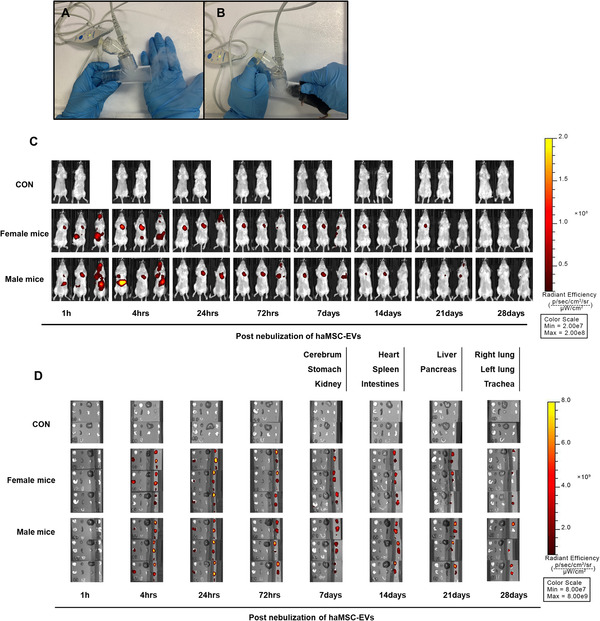
Mesh nebulizer set‐up and biodistribution of haMSC‐EVs. (a‐b) Vibrating mesh nebulizer set‐up. (c) Biodistribution of DiR‐labelled haMSC‐EVs *in vivo* continuously until 28 days post‐nebulization. (d) Biodistribution of DiR‐labelled haMSC‐EVs *in vitro* continuously until 28 days post‐nebulization. The list of terms represents the layout of isolated organs. haMSC‐EVs: human adipose‐derived MSC‐Extracellular vesicles

The mice were nebulized with either saline (1 ml) or different amounts of haMSC‐EVs (2 × 10^5^, 6 × 10^5^, 10^6^, 2 × 10^6^, 6 × 10^6^, 10^7^ particles) diluted in saline (1 ml), 2 h after *P. aeruginosa* intratracheal instillation. The *P. aeruginosa‐*induced pneumonia model has been previously described by our team (Shi et al., 2021). For the nebulization process, starting from the morning of the experimental day, inhalation of haMSCs‐EVs was administered using a vibrating mesh nebulizer set (Aerogen Solo system, Ireland) with a total volume of 1 ml solution for 5 min (Figure 2a, b). Survival rates were observed until 96 h.

haMSC‐EVs were labelled fluorescently with the cell membrane dye, DiR (1,1‐dioctadecyl‐3,3,3,3‐tetramethylindotricarbocyanine iodide) (Invitrogen, USA). BALB/c mice were nebulized with haMSC‐EVs (6 × 10^8^ particles per mouse) and then continuously observed the biodistribution for up to 28 days. The mice were sacrificed at each time point respectively post‐administration. Different organs such as the cerebrum, stomach, kidney, heart, spleen, intestines, liver, pancreas, lung and trachea were also collected to measure the fluorescence intensity *in vitro*.

In separate experiments, mice were inoculated intratracheally with 10^6^ CFU of *P. aeruginosa* strain and were then treated 2 h later with nebulized with 1 ml saline, haMSC‐EVs (2 × 10^6^ particles), or extracellular vesicles derived from 929 cells (L‐929 EVs) (6 × 10^6^ particles) as a negative control. Both bronchoalveolar lavage fluid (BALF) samples and lungs were collected 24 h post‐infection. According to the manufacturer's protocols, total and differential cell counts in BALF were obtained using Sysmex pocH‐100i (Sysmex Shanghai Ltd., China). BALF samples were centrifuged at 3000 rpm for 10 min, and the supernatants were collected and stored at ‐20°C. Interleukin‐6 (IL‐6), Tumour necrosis factor‐α (TNF‐α), and Interleukin‐10 (IL‐10) were measured by ELISA (R&D Systems, Minneapolis, Minnesota, USA) in the BALF supernatants. The lungs were gently inflated with 0.5 ml of 4% formalin, followed by tracheal ligature, fixed in 4% Formalin, and dehydrated through serial diluted graded ethyl alcohol baths. After fixation, lungs were embedded in paraffin, cut into 5 μm sections, and stained with Haematoxylin & Eosin. Levels of lung injury were assessed using the lung injury score (LIS), which varies from 0 to 1, and a higher score of which means more injury. An independent investigator, blinded to group assignments, assessed whether lung parenchyma was infected or not. Pneumonia was defined as the presence of polymorphonuclear neutrophils (PMNs) within septa and alveolar spaces. The severity of lung injury was determined according to the scoring system of the American Thoracic Society (Shi et al., 2019).

### 2.6 Design of the phase 1 single‐arm clinical trial MEXVT

The safety and tolerance of nebulized haMSC‐EVs were observed among healthy volunteers in a phase 1 single‐arm clinical trial (**MEXVT** study, **NCT04313647**). The Rui‐jin Hospital Ethics Committee, Shanghai Jiao‐tong University School of Medicine approved this study (2020‐36‐3), following the declaration of Helsinki. All volunteers were enrolled in Rui‐jin hospital, Shanghai Jiao‐tong University School of Medicine, Shanghai, China. As the preclinical study showed that the optimal therapeutic dosage in mice was 10^5^ particles/g, we determined the testing dose started from 2 × 10^8^ particles based on our previous murine experiments. MEXVT clinical trial is an open‐label, dose‐escalation pilot study in which five cohorts of healthy volunteers received increasing doses of haMSC‐EVs administered as a single nebulization. There are three or six subjects per cohort, with patients in each cohort receiving either 2.0 × 10^8^ particles (first cohort), 4.0 × 10^8^ particles (second cohort), 8.0 × 10^8^ particles (third cohort), 12.0 × 10^8^ particles (fourth cohort) or 16.0 × 10^8^ particles (fifth cohort).

### 2.7 Clinical trial procedures

Informed consent was obtained after discussion with the healthy volunteer. The MEXVT study flow is summarized in Figure 4. Supplementary Panel 1 shows the eligibility criteria. haMSC‐EVs were prepared from CBMG and shipped frozen to the clinical site in a validated dry ice shipper with a continuous temperature monitoring device. Upon receipt, the EV solution was inspected and stored in a controlled, continuously monitored ‐20°C storage tank. Prior to administration, the solutions were thawed, reconstituted at the clinical site. Data from the first volunteer of the first cohort were reviewed for safety before proceeding with an enrolment of the following volunteers or escalation of the dose. The protocol included a provision that the Data Safety Monitoring Group (DSMG) or the study sponsor could decide to enrol more healthy volunteers at any dose level if there were any prespecified inhalation‐associated adverse events or serious adverse events related to haMSC‐EVs. Medical history, clinical characteristics and laboratory measurements were collected when every healthy volunteer was enrolled and admitted on Day 0. The skin test of haMSC‐EVs was performed before the first nebulization in order to avoid potential allergic reactions. The haMSC‐EVs solution was thawed at room temperature and was shaken to make it completely suspended. The skin test solution was composed of 0.1 ml haMSC‐EVs diluted in 0.9 ml normal saline. After checking that there was no skin test contraindication, the nurse injected 0.1 ml skin test solution intradermally on the palm of the forearm (3 cm above the wrist). The skin test result was observed after 20 min, including negative (no reaction at the skin test site, no wheal with itching, no papules, no urticaria, no redness and swelling around) and positive (obvious wheals, papules or urticaria appeared, peripheral congestion and swelling, blisters, itching, etc.).

On the morning of Day 1, single nebulization of haMSC‐EVs was initiated using a mesh nebulizer set (Aerogen Solo system, Ireland) with a total volume of 6 ml diluted with normal saline for 30 min. The physician investigator remained at the bedside for the nebulization duration and 24 h after the inhalation was initiated to observe any signs of an adverse reaction. Follow‐up laboratory tests such as blood routine test, liver and renal function, lactate dehydrogenase, immunoglobulins and cardiogram were collected on Day 7 for safety monitoring.

### 2.8 Statistical analysis

The laboratory parameter values were compared using the Kruskal‐Wallis test. The data of animal experiments were shown as mean ± SEM. The log‐rank test was used for comparing the survival rate at 96 h. Other continuous variables were presented as median and 25–75^th^ interquartile range (IQR). All statistical analysis was performed using GraphPad Prism software (La Jolla, CA, USA). The remaining analyses are descriptive.

## 3 RESULTS

### 3.1 The characterization of haMSC‐EVs and the biodistribution of nebulized haMSC‐EVs in murine model

As shown in Figure 1b, the morphologies of haMSC‐EVs were observed by transmission electron microscopy examination. The results of NTA showed that the size distribution of haMSC‐EVs was 50–400 nm in diameter (Figure 1c). The haMSC‐EVs were enriched in the specific markers, including CD9, CD81 and CD63. Both haMSC and haMSC‐EVs expressed TSG101, while haMSCs were enriched in CANX (Figure 1d, Figure S1). Also, the characteristics of L‐929‐derived EVs were shown in Figure S2 and the uncut western blots of L‐929‐derived EVs were shown in Figure S3.

We then used DiR‐labelled haMSC‐EVs to investigate the biodistribution of haMSC‐EVs administered *in vivo* either through the intravenous or nebulized route. Mice were exposed to nebulized haMSC‐EVs or normal saline (NS) using a vibrating mesh nebulizer set (Figure 2a, b). The strongest fluorescence intensity at 24 h post‐nebulization appeared in the lung, and the one at 24 h remained to be detected within the stomach. Meanwhile, the fluorescence intensity in the lung gradually decreased up to 28 days after nebulization (Figure 2c, d).

### 3.2 Therapeutic effects of haMSC‐EVs on survival rate, lung inflammation, protein permeability and histological severity in *P. aeruginosa*‐induced lung injury

**FIGURE 3:**
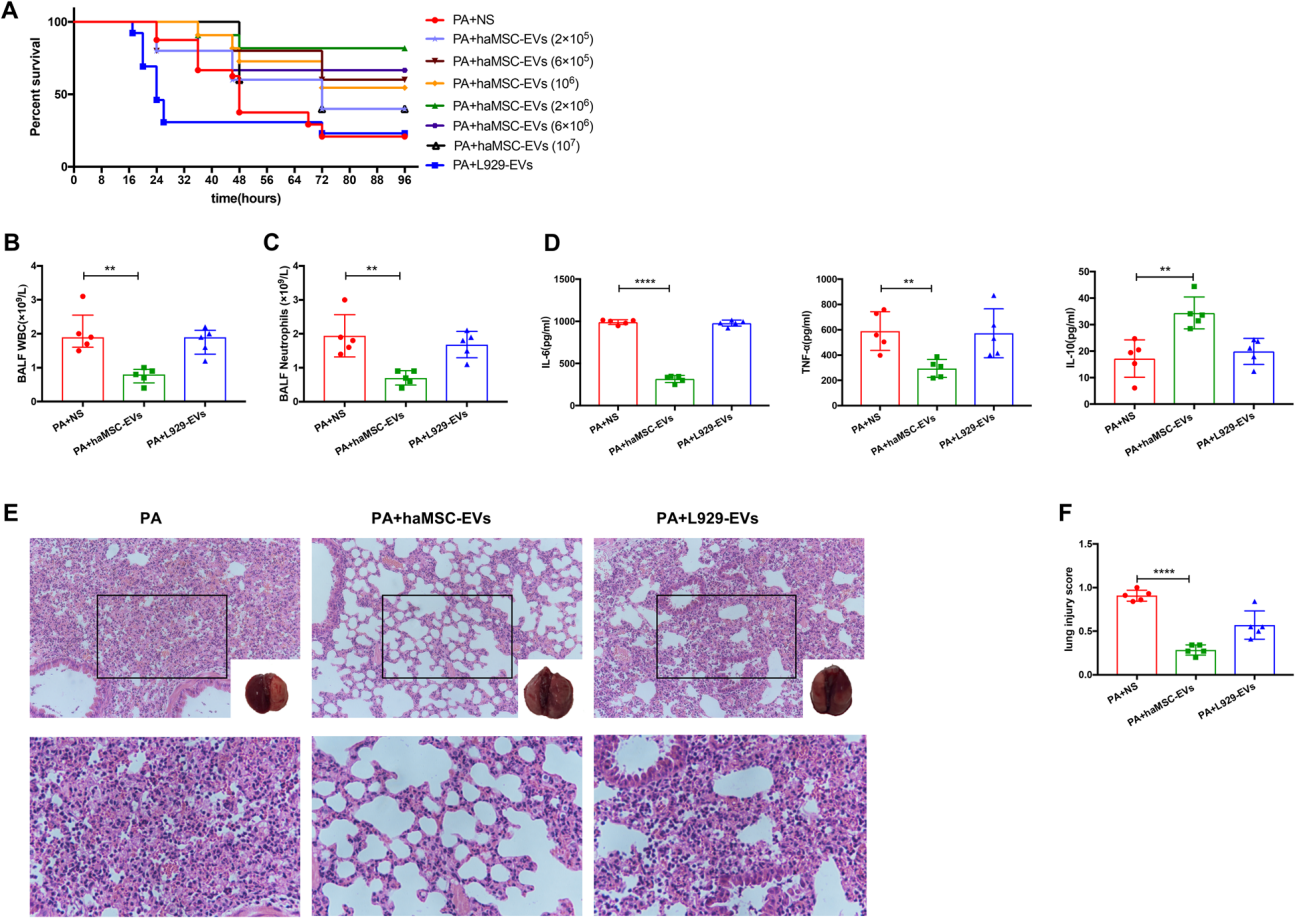
Therapeutic effects of haMSC‐EVs in *P. aeruginosa‐*induced murine lung injury model. (a) Kaplan‐Meier survival curves of *P. aeruginosa*‐induced murine lung injury model (N = 10 per group). (b‐c) Aerosol inhalation of haMSC‐EVs post‐infection decreased the influx of white blood cells (*P* = 0.0026, ** indicates *P* < 0.01, 2.0 ± 0.3 for PA + NS, 0.8 ± 0.1 for PA + haMSC‐EVs, N = 5) and neutrophils (*P* = 0.0029, ** indicates *P* < 0.01, 1.9 ± 0.3 for PA, 0.7 ± 0.1 for PA + haMSC‐EVs, N = 5) in BALF at 24 h. (d) The BALF levels of IL‐6 (**** indicates *P* < 0.0001, 990.1 ± 12.3 for PA + NS, 316.3 ± 18.9 for PA + haMSC‐EVs, N = 5), TNF‐α (*P* = 0.0044, ** indicates *P* < 0.01, 589.9 ± 68.4 for PA + NS, 294.8 ± 31.7 for PA + haMSC‐EVs, N = 5) were decreased in haMSC‐EVs group. The level of IL‐10 expression was increased (*P* = 0.0032, ** indicates *P* < 0.01, 17.2 ± 3.1 for PA + NS, 34.4 ± 2.7 for PA + haMSC‐EVs, N = 5) in haMSC‐EVs group. (e) The histology showed less inflammatory cells infiltrating interalveolar septa and respecting alveolar space and lung architecture. (f) Aerosol inhalation of haMSC‐EVs post‐infection also reduced histological severity of lung injury better than other groups (**** indicates *P* < 0.0001, 0.908 ±0.03 for PA + NS, 0.284 ± 0.03 for PA + haMSC‐EVs, N = 5). haMSC‐EVs: human adipose‐derived MSC‐Extracellular vesicles; PA: *P. aeruginosa*; L‐929‐Exos: NCTC clone 929 cells derived exosomes; BALF: bronchoalveolar lavage fluid; WBC: white blood cell; IL‐6: interleukin 6; TNF‐α: tumour necrosis factor‐α; IL‐10: interleukin 10

Our previous study showed that intratracheally instillation of 10^7^ CFU of *P. aeruginosa* resulted in lethal pneumonia, leading to 80% of mortality at 96 h (Shi et al., 2021). Nebulized haMSC‐EVs (10^6^ particles) increased survival to 60% at 96 h compared with mice treated with NS as the negative control. Inhalation of haMSC‐EVs (2 × 10^6^ particles) further increased survival to 80% at 96 h. The survival at both of the two haMSC‐EVs groups was significantly higher than the NS group (*P *< 0.01), as well as the negative control, the NCTC clone 929 cells derived EVs (L929‐EVs) group (Figure 3a).

Intratracheally instilling 10^6^ CFU *P. aeruginosa* induced non‐lethal but robust pneumonia‐induced acute lung injury over 24 h, according to our previous study (Shi et al., 2021). Based on the survival rate, doses of 2 × 10^6^ particles of haMSC‐EVs were selected for the following *in vivo* experiments. Inhalation of haMSC‐EVs reduced the influx of bronchoalveolar lavage fluid (BALF) white blood cells (WBC) by 59% (2.0 ± 0.3 vs. 0.8 ± 0.1 × 10^9^/L, *P *= 0.0026), and neutrophils by 60% (1.9 ± 0.3 vs. 0.7 ± 0.1 × 10^9^/L, *P *= 0.0029), as compared with inhalation of NS only (Figure 3b, c). Similarly, nebulized haMSC‐EVs decreased the levels of IL‐6 by 67% (990.1 ± 12.3 vs. 316.3 ± 18.9 ρg/ml, *P *< 0.0001), of TNF‐α by 48% (589.9 ± 68.4 vs. 294.8 ± 31.7 ρg/ml, *P *= 0.0044), while increasing the levels of IL‐10 by 94% (17.2 ± 3.1 vs. 34.4 ± 2.7 ρg/ml, *P *= 0.0032) at 24 h, as compared with NS (Figure 3d). Meanwhile, a similar beneficial effect on the pulmonary macroscopic and histologic injury was also observed in mice receiving treatment of haMSC‐EVs (Figure 3e, f).

### 3.3 General characteristics of MEXVT

From 12 March 2020, to 31 July 2020, a total of 26 healthy volunteers (13 males and 13 females) were screened for eligibility, and two of them were excluded from this study, with one of positive skin test (shown in Figure S4) and one of the elevated levels of WBC counts. Eventually, 24 healthy volunteers (13 males and 11 females) received nebulized haMSC‐EVs. Baseline demographic characteristics for the 24 subjects are shown in Table S1.

### 3.4 Safety of nebulized haMSC‐EVs in all cohorts of healthy volunteers

**FIGURE 4:**
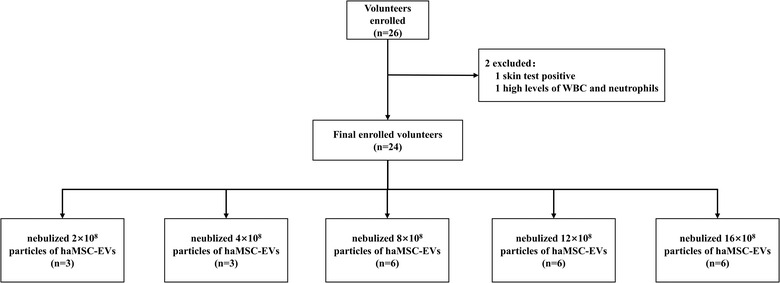
Flow diagram for MEXVT. WBC: white blood cell; haMSC‐EVs: human adipose‐derived MSC‐Extracellular vesicles

**FIGURE 5:**
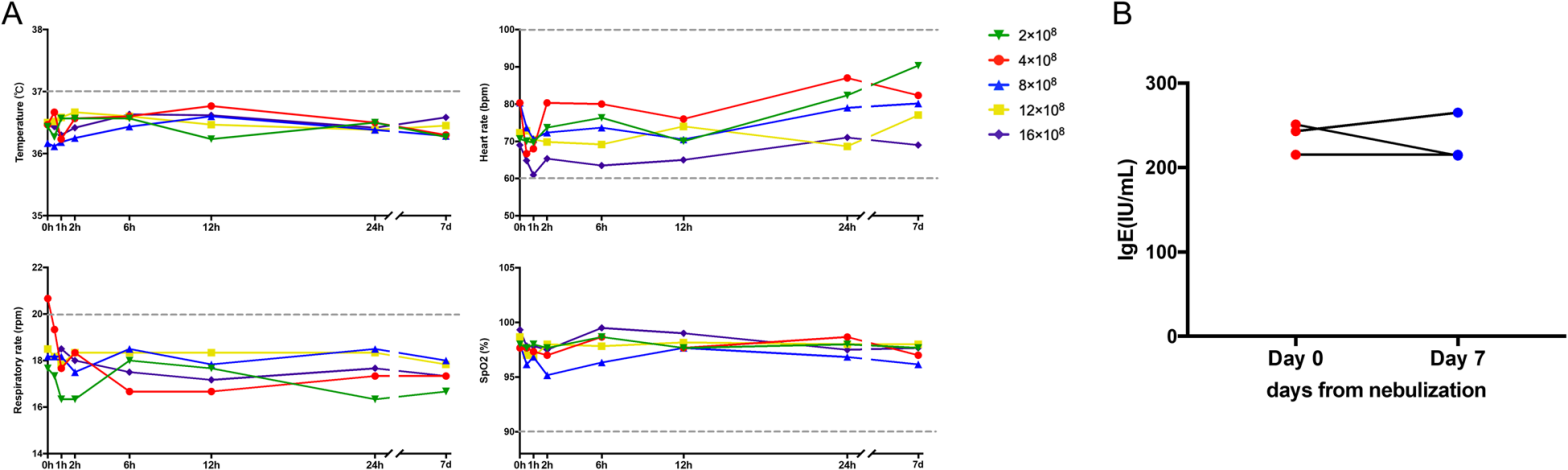
Clinical characters of healthy volunteers. (a) Vital sign parameters before and after haMSC‐EVs nebulization in MEXVT. (b) Level of IgE before and after haMSC‐EVs nebulization in MEXVT. haMSC‐EVs: human adipose‐derived MSC‐Extracellular vesicles; IgE: ImmunoglobulinE

There were twenty‐four volunteers who received haMSC‐EVs nebulization (Figure 4). All volunteers tolerated the haMSC‐EVs nebulization well. No significant changes in vital signs (temperature, heart rate, respiratory rate and saturation oxygen) and laboratory parameters were reported among volunteers in all groups during the nebulization or in the 7‐day follow‐up period (Figure 5a, Table S2). Specifically, three of the volunteers had elevated baseline levels of IgE. After inhalation of haMSC‐EVs, the IgE levels decreased in one of them (Figure 5b). Two volunteers (2/24, 8.3%) presented sinus bradycardia in EKG on the seventh day of nebulization: one (heart rate: 58 bpm) was in the group of 2 × 10^8^ particles, the other (heart rate: 59 bpm) was in the group of 16 × 10^8^ particles. One volunteer had a low basal heart rate (heart rate: 58 bpm), another volunteer's basal heart rate was 70 bpm. The relative bradycardia of the two volunteers did not impact the blood pressure and both of them were hemodynamically well‐tolerated. No other adverse events were reported, and no serious adverse events were observed from starting nebulization to the 7th day after nebulization. No allergy to haMSC‐EVs was reported among them (Table S2).

## 4 DISCUSSION

Various preclinical studies have demonstrated that intravenous administration of MSC‐EVs is a promising source of cell‐free therapy for infectious lung injury, including influenza virus‐induced ALI (Khatri et al., 2018; Loy et al., 2019) and bacteria‐induced severe pneumonia in mice (Curley et al., 2017; Devaney et al., 2015; Gupta et al., 2012; Kim et al., 2011; Lee et al., 2013; Mao et al., 2015; Monsel et al., 2015; Park et al., 2019; Shi et al., 2021; Sung et al., 2016). Few studies have explored the nebulized MSC‐EVs for pulmonary disease. *McCarthy* et al. reported that through vibrating mesh nebulization delivery, MSC‐derived conditioned medium retained their bactericidal properties against *E. coli*, *Staphylococcus aureus*, and multidrug‐resistant *Klebsiella pneumoniae* clinical isolates (McCarthy et al., 2020). *Phuong‐Uyen C. Dinh* et al. presented a series of studies utilizing lung spheroid cell‐secretome (LSC‐Sec) and exosomes (LSC‐Exo) by inhalation to treat different models of lung injury and fibrosis (Dinh et al., 2020). We used vibrating mesh nebulizers in all our trials because there is a body of evidence supporting the use of mesh rather than jet nebulizers (Galindo‐Filho et al., 2019; Rouby et al., 2020; Zhou et al., 2014). Mass median aerodynamic diameters were slightly greater with mesh nebulizers compared to jet nebulizers but always remained below five microns, ensuring a passing of the nebulized product beyond the 23^rd^ generation of bronchiolar subdivision, which is required to reach the site of lung injury. Biodistribution of DiR‐labelled haMSC‐EVs *in vivo* showed that the lung's fluorescence intensity peaked at 24 h post nebulization and then gradually decreased up to 28 days. Interestingly, the intensity remained to be detected within the stomach at 24 h. A systemic diffusion of labelled EVs might be explained by the possibility of EVs swallowing during the nebulization process, similar to a kind of “oral route”. EVs systemic diffusion from the lungs is still possible and requires future investigations. In addition, the nebulized route avoids the trapping of vesicles in the liver and spleen, commonly reported during intravenous administration of vesicles. In this study, nebulized haMSC‐EVs improved survival rate to 80% at 96 h in the *P. aeruginosa*‐induced pneumonia model of mice by decreasing lung inflammation and histological severity, consistent with our previous study in which MSCs‐microvesicles exhibited protective effects in severe bacterial pneumonia (Monsel et al., 2015). The findings supported that haMSC‐EVs nebulization 2 h after bacterial inoculation was effective in attenuating lung damage. We as well observed a dose‐response effect of haMSC‐EVs in the murine lung injury model: within the dose range of 2 × 10^5^ to 2 × 10^6^ particles, higher dosage resulted in more favourable survival. However, in groups with higher dosages beyond 2 × 10^6^ particles, the dosage was negatively correlated with survival rate. Such a phenomenon suggested that the appropriate dosage is a prerequisite for haMSC‐EVs’ beneficial impact.

The therapeutic potential of MSCs and their derived extracellular vesicles have been widely studied, whereas the related clinical trials are rare as a number of studies are still in the preclinical stage. In a phase 1 clinical trial, the intravenous infusion of bone marrow‐derived human MSCs was well tolerated in nine patients with moderate to severe ARDS (Wilson et al., 2015). A randomized, placebo‐controlled pilot study involving 12 adult patients with ARDS demonstrated that administration of allogeneic adipose‐derived MSCs was safe and feasible in treating ARDS without infusion toxicities or serious adverse events (Zheng et al., 2014). Another phase 2a trial showed that intravenous 10 × 10^6^ MSCs/kg was safe in patients with moderate to severe ARDS (Matthay et al., 2019). Three studies suggested that intravenous administration of MSC and MSC‐EVs were promising therapeutic candidates for severe lung injury caused by SARS‐CoV‐2, owning to their safety and capability to improve oxygenation and alleviate inflammation (Meng et al., 2020; Sengupta et al., 2020; Shu et al., 2020). However, the administration route is likely to be one of the underlying mechanisms of the insufficient effect of applying MSC‐EVs intravenously in published articles. Since a significant amount of loss of EVs may occur after intravenous administration due to rapid uptake by endothelial cells after injection, the biodistribution of haMSC‐EVs in our study suggested that the nebulized route would be the more promising route. Thus, the clinically scientific and ethically correct approach seems to be the nebulized local application in order to achieve a high concentration of substances at the site of therapeutic activity in respiratory diseases. Our study is subsequently designed to evaluate the safety of clinical‐grade haMSC‐EVs nebulization in healthy volunteers. As the human immune system and its response to external stimuli were more complex, the mass dosage equivalence did not necessarily apply when translating from rodents to humans. For maximum safety, we started from a 10‐fold reduction and gradually increased to 1.6 × 10^9^. Coinciding with previous articles on MSCs for other clinical indications (Bartolucci et al., 2017; Gao et al., 2016; Panés et al., 2016; Petrou et al., 2016), nebulized haMSC‐EVs performed favourable tolerability and short‐term safety profile. Based on these encouraging results, a multicentre, randomized, double‐blind, placebo‐controlled clinical study of allogeneic human adipose‐derived mesenchymal stromal cell extracellular vesicles (haMSC‐EVs) nebulizer for the treatment of ARDS is in progress (**NCT04602104**).

This study suffers from some potential limitations. First, several industry criteria such as high throughput production of MSC for scaling‐up the EVs production as well as inter batch consistency remain standardized for future clinical application. Second, few potency assays are available for the final products. All the existing evidence comes from the mice model, which may have discrepancies in the process of translation from bench to bedside.

In conclusion, nebulized haMSC‐EVs exerted protective effects in severe pneumonia caused by *P. aeruginosa* in mice, and nebulized haMSC‐EVs were safe in healthy volunteers without serious side effects. This study indicates that nebulized haMSC‐EVs could be a promising therapeutic strategy and offers preliminary evidence to promote the future clinical applications of nebulized haMSC‐EVs in lung injury diseases.

## CONFLICT OF INTEREST

The authors indicate no potential conflicts of interest.

## AUTHOR CONTRIBUTIONS

Ying‐Gang Zhu and Jie‐Ming Qu designed the trial. Meng‐Meng Shi, Qing‐Yuan Yang, Jia‐Yang Yan, Antoine Monsel, Cheng‐Xiang Dai, Su‐Ke Li, Ping Li, Jing‐Ya Zhao, Guo‐Chao Shi, Min Zhou, Xue‐Mei Zhu, Jing Wang, Meng Li, Ji‐Gang Lei, and Xu Dong collected data. Meng‐Meng Shi, Qing‐Yuan Yang, Jia‐Yang Yan, Ying‐Gang Zhu analysed data. Meng‐Meng Shi, Antoine Monsel, Ying‐Gang Zhu, Jie‐Ming Qu interpreted data. Meng‐Meng Shi, Qing‐Yuan Yang, Jia‐Yang Yan and Ying‐Gang Zhu wrote the report. Meng‐Meng Shi, Qing‐Yuan Yang, and Ying‐Gang Zhu prepared the figures. Antoine Monsel, Cheng‐Xiang Dai, Ying‐Gang Zhu, Jie‐Ming Qu provided editorial overview and modified the report.

## Supporting information

Supporting Information.
