# Supplementary material for: Preclinical efficacy and clinical safety of clinical‐grade nebulized allogenic adipose mesenchymal stromal cells‐derived extracellular vesicles
Source: J Extracell Vesicles. 2021 Aug 14;10(10):e12134. doi: 10.1002/jev2.12134 (PMC8363910; doi:10.1002/jev2.12134)
Supplement: Supplementary file 1 — Supporting Information. [file JEV2-10-e12134-s001.docx]

**SUPPLEMENTARY MATERIAL**

**Preclinical Efficacy and Clinical Safety of Clinical-Grade Nebulized Allogenic Adipose Mesenchymal Stromal Cells-Derived** **Extracellular Vesicles**

Meng-meng Shi^#^ MD^1,2,3^; Qing-yuan Yang^#^ MD^1,2,3^; Antoine Monsel^#^ MD, PhD^4,5,6^; Jia-yang Yan^#^ MD^1,2,3^; Cheng-xiang Dai^#^ MD, PhD^7,8^; Jing-ya Zhao MD^1,2,3^; Guo-chao Shi MD, PhD^1,2,3^; Min Zhou MD, PhD^1,2,3^, Xue-mei Zhu MD, PhD^1,2,3^; Su-ke Li, MS^7^; Ping Li, MS^7^; Jing Wang, PhD^7^; Meng Li, MS^7^; Ji-gang Lei, PhD^7^; Dong Xu, MS^7^; Ying-gang Zhu* MD, PhD^9^; Jie-ming Qu* MD, PhD^1,2,3^

**Affiliations:**

^1^Department of Pulmonary and Critical Care Medicine, Rui-jin Hospital, Shanghai Jiao-tong University School of Medicine, Shanghai, China

^2^Institute of Respiratory Disease, Shanghai Jiao-tong University School of Medicine, Shanghai, China

^3^Key Laboratory of Emergency Prevention, Diagnosis and Treatment of Respiratory Infectious Diseases, Shanghai, China

^4^Multidisciplinary Intensive Care Unit, Department of Anesthesiology and Critical Care, La Pitié-Salpêtrière Hospital, Assistance Publique-Hôpitaux de Paris (APHP), Sorbonne University, France

^5^Sorbonne Université, INSERM, UMR S 959, Immunology-Immunopathology- Immunotherapy (I3); F-75005, Paris, France

^6^Biotherapy (CIC-BTi) and Inflammation-Immunopathology-Biotherapy Department (DHU i2B), Hôpital Pitié-Salpêtrière, AP-HP, F-75651, Paris, France

^7^Cellular Biomedicine Group Inc. (CBMG), Shanghai, China

^8^Daxing Research Institute, University of Science and Technology Beijing, Beijing, China

^9^Department of Pulmonary and Critical Care Medicine, Hua-dong Hospital, Fudan University, Shanghai, China

**#Authors contributed equally**

***Address correspondence to:**

Jie-Ming Qu, MD, PhD

Rui-jin Hospital, Shanghai Jiao-tong University School of Medicine

Department of Pulmonary and Critical Care Medicine

197, Rui Jin Er Rd.

Shanghai, China, 200025

Telephone: +86-21-64370045

Email: jmqu0906@163.com

OR

Ying-Gang Zhu, MD, PhD

Hua-dong Hospital, Fudan University

Department of Pulmonary and Critical Care Medicine

221, West Yan’an Rd.

Shanghai, China, 200040

Telephone: +86-21-62483180

Email: robinzyg@gmail.com

**Supplementary Material include:**

**Supplement Methods:**

Detailed information of haMSCs used in this study

**Supplementary Panel 1.** The eligibility criteria of the MEXVT study.

**Supplement Figures:**

**Supplementary Figure 1.** Uncut western blots of haMSC-EVs.

**Supplementary Figure 2.** The characteristics of L-929-derived EVs.

**Supplementary Figure 3.** Uncut western blots of L-929-derived EVs.

**Supplementary Figure 4.** A Representative of positive skin test.

**Supplement Tables:**

**Supplementary Table 1.** Baseline characteristics of healthy volunteers in MEXVT

**Supplementary Table 2.** Laboratory parameters before and after haMSC-EVs nebulization in MEXVT

**Supplementary methods**

***Detailed information of haMSCs used in this study***

The haMSCs used in this clinical study were 1 batch derived from 1 donor. The healthy young adult donors signed the informed consent reviewed by the ethics committee in the teaching hospital and finished the various screening tests, including physical check and the serum virus tests on HIV-1, HIV-2, HCV, HBV, Syphilis, HTLV-1, HTLV-2, and CMV.

The master cell bank (P1) and working cell bank (P4) have fulfilled strict quality control tests, including: 1) negative virus tests on CMV, HIV-1, HIV-2, HTLV-1, HTLV-2, EBV, HBV and HCV; 2) negative bacterial, fungal and mycoplasma; 3) endotoxin ≤100EU/mL; 4) cell viability >90%; 5) surface marker expression of ≥95% CD73^+^, CD90^+^, CD105^+^, and ≤2% CD34^-^, CD45^-^, HLA^-^DR^-^. Cell morphology, viability, colony-forming efficiency, differentiation potency, and bovine viruses have also been tested in the master cell bank.

**Supplementary Panel 1.** The eligibility criteria of the MEXVT study.

**Inclusion criteria:**

1. The subjects voluntarily participated in the study and signed the informed consent.

2. Healthy volunteers.

3. Ages ranged from 19 to 45 years old.

4. Normal routine laboratory parameters.

5. According to the Good Clinical Practice (GCP), volunteers voluntarily participated in this study and signed the informed consent.

**Exclusion criteria:**

1. Pregnant or nursing females.

2. Suffering from major diseases.

3. Mentally or physically disabled.

4. Alcohol or drug abuse.

5. People who are taking some therapeutic drugs.

6. Allergy history of more than 2 drugs or food.

7. According to the judgment of the researcher, the one who has a low probability of being included in the group (such as frailty, etc.).

**Supplementary Figures**
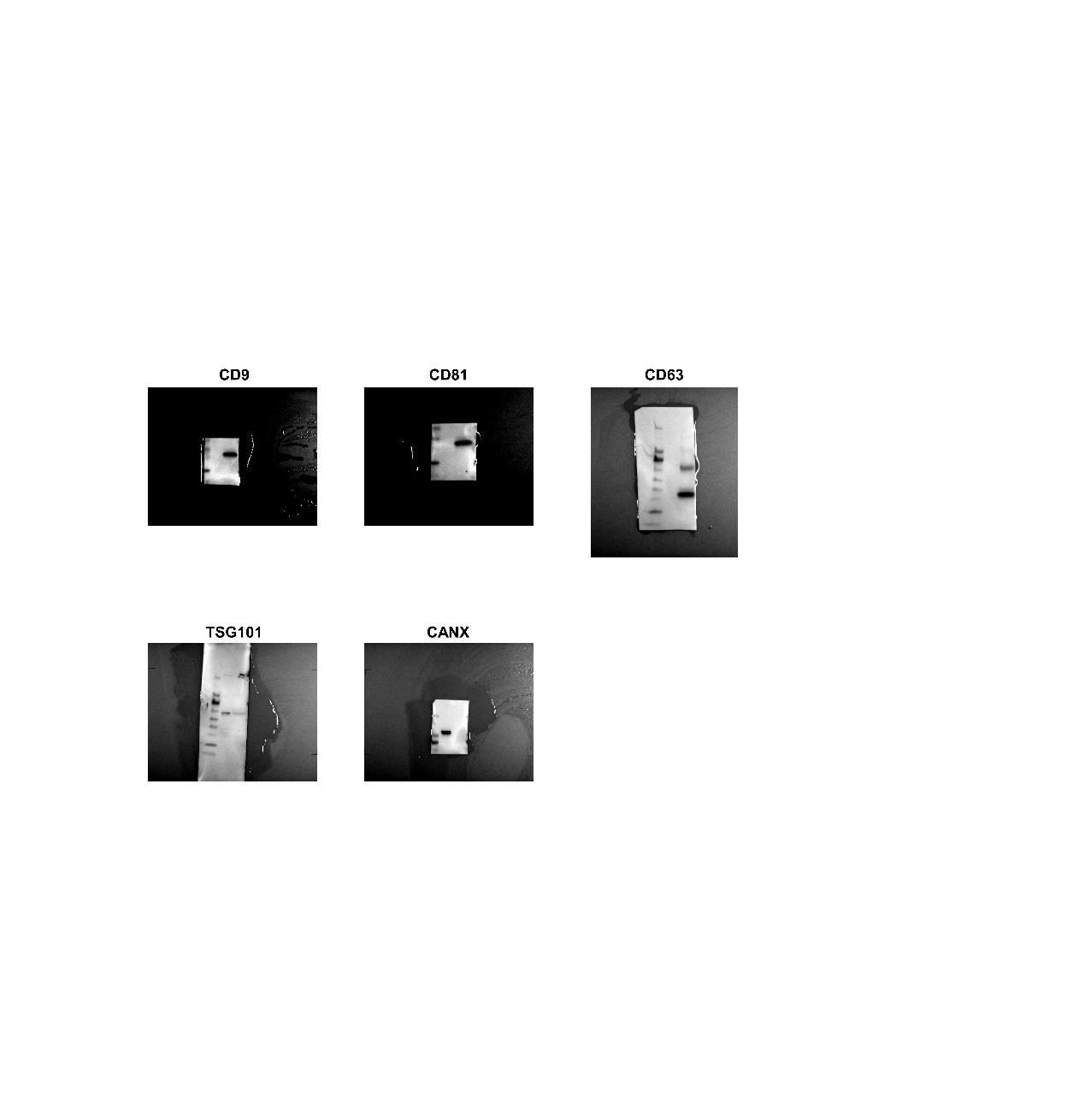


**Supplementary Figure 1.** Uncut western blots of haMSC-EVs. Uncut western blots showing the expression of haMSC-EV markers, including CD9, CD81, CD63, TSG101, and CANX.


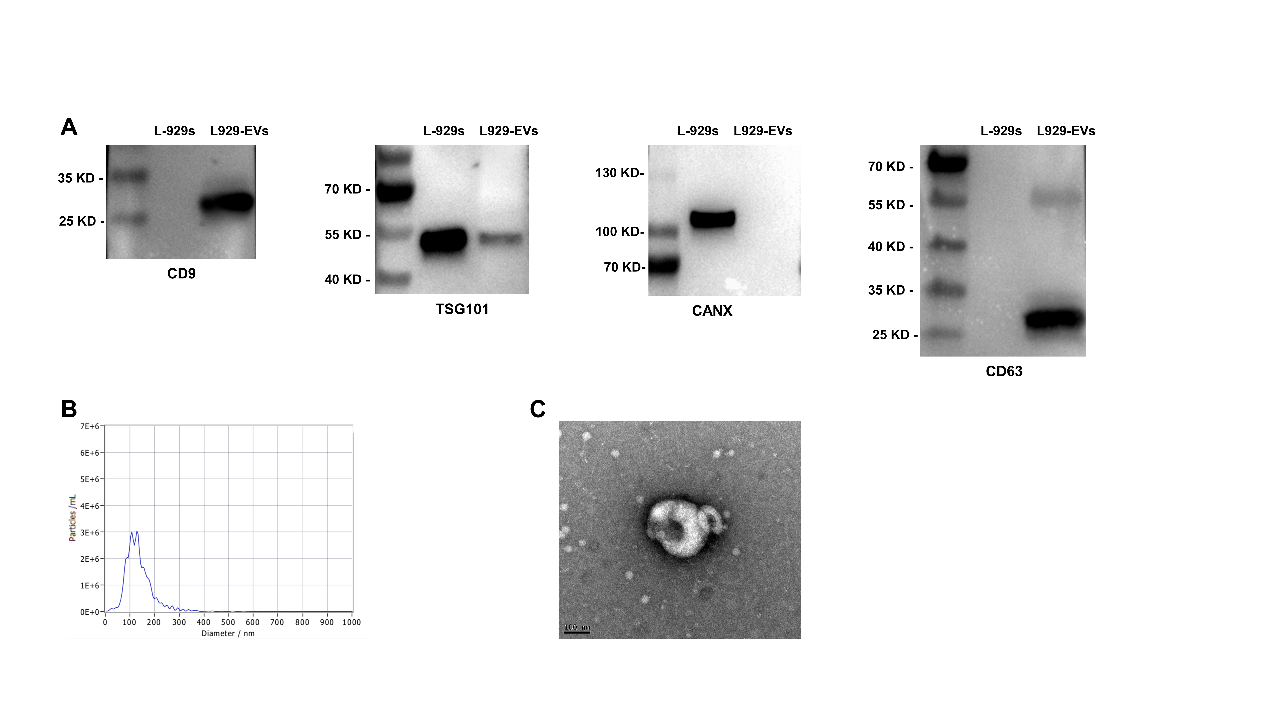


**Supplementary Figure 2.** The characteristics of L-929-derived EVs**. (A)** Representative western blots showed the expression of EV markers, including CD9, TSG101, CANX, and CD63. **(B)** The concentration and size distribution of L-929-EVs were determined by NTA. **(D)** Representative electron microscopic photograph of L-929-EVs, scale bar =100 nm.

L-929-derived EVs: L-929 derived-Extracellular vesicles; NTA: Nanoparticle Tracking Analysis.


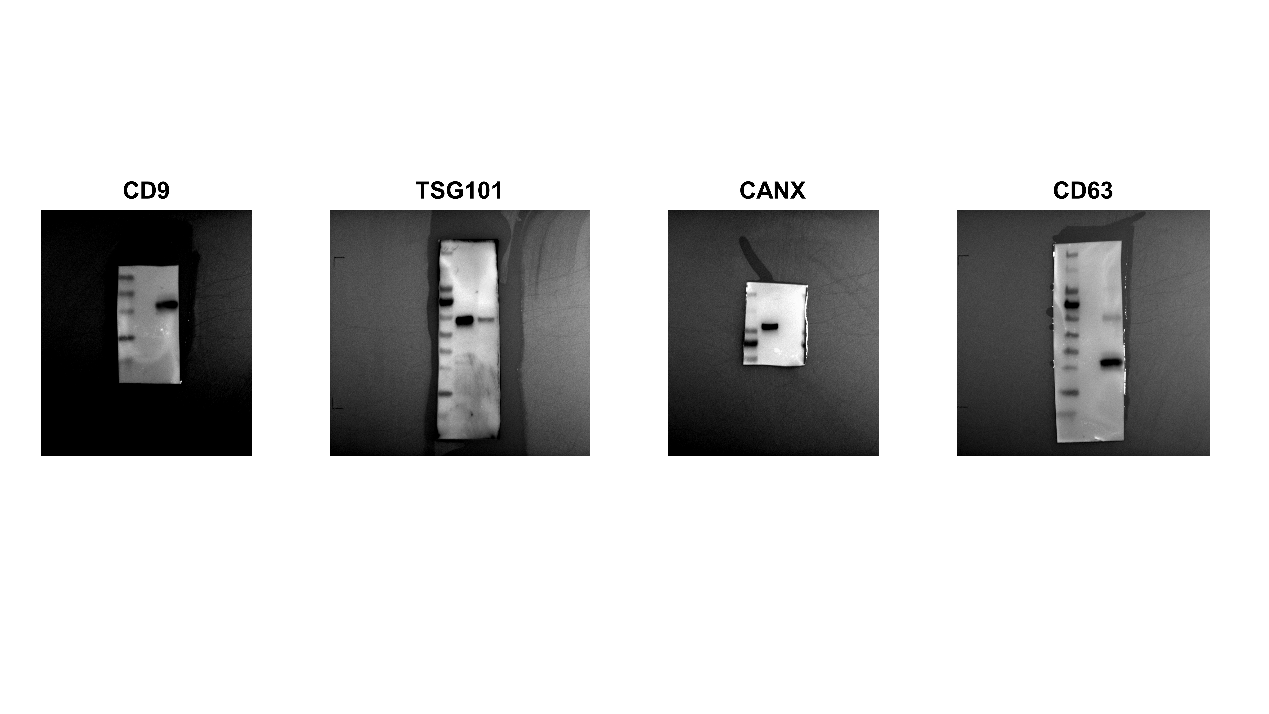


**Supplementary Figure 3.** Uncut western blots of L929-derived EVs. Uncut western blots showing the expression of L-929-derived EV markers, including CD9, TSG101, CANX, and CD63.

L-929-derived EVs: L-929 derived-Extracellular vesicles


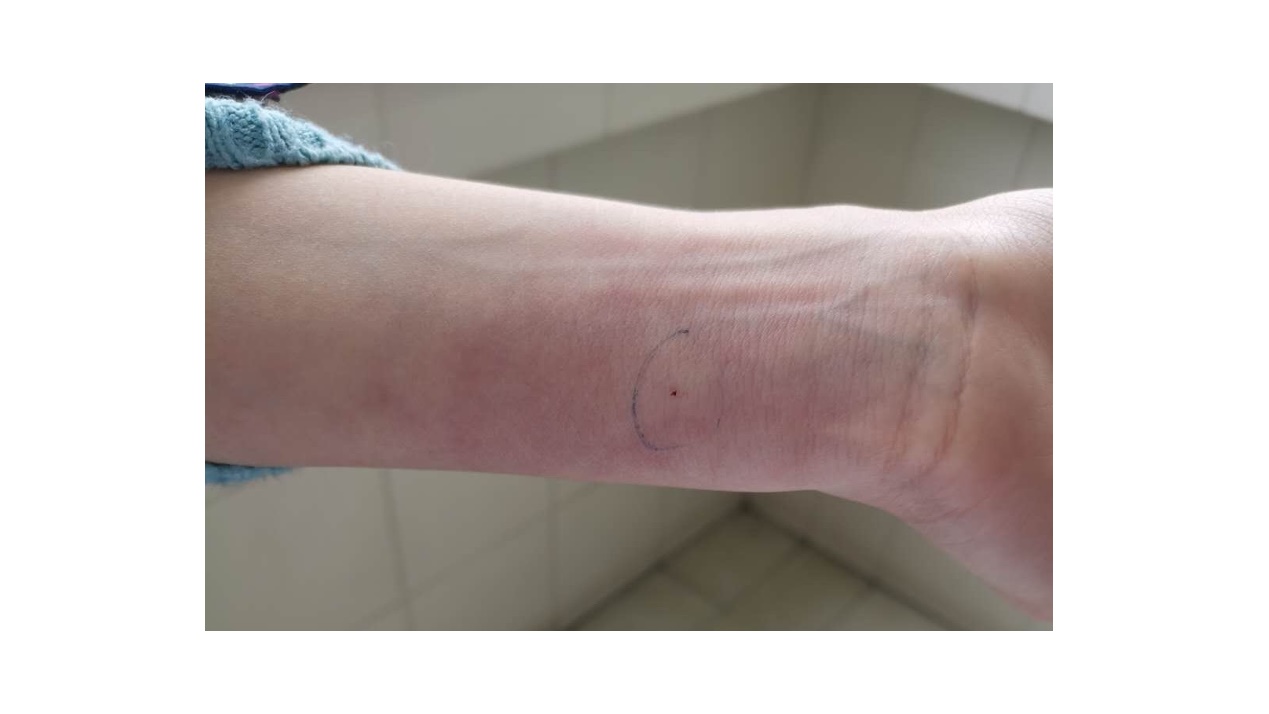


**Supplementary Figure 4.** A Representative of positive skin test.

**Supplementary Table 1. Baseline characteristics of healthy volunteers in MEXVT**

|  | Age  (years) | Sex | BMI | Allergic history | Comorbidity | Smoking history |
| --- | --- | --- | --- | --- | --- | --- |
| 2×10^8^ particles | | | | | | |
| Volunteer 1 | 25 | Male | 22.491 | NA | NA | NO |
| Volunteer 2 | 26 | Female | 26.235 | NA | NA | NO |
| Volunteer 3 | 34 | Female | 23.875 | NA | NA | NO |
| 4×10^8^ particles | | | | | | |
| Volunteer 4 | 27 | Male | 29.061 | NA | NA | NO |
| Volunteer 5 | 26 | Male | 22.093 | NA | NA | NO |
| Volunteer 6 | 24 | Female | 19.922 | NA | NA | NO |
| 8×10^8^ particles | | | | | | |
| Volunteer 7 | 25 | Female | 19.628 | NA | NA | NO |
| Volunteer 8 | 35 | Male | 25.952 | NA | NA | NO |
| Volunteer 9 | 25 | Female | 19.706 | NA | NA | NO |
| Volunteer 10 | 21 | Male | 19.818 | NA | NA | NO |
| Volunteer 11 | 31 | Male | 25.352 | NA | NA | NO |
| Volunteer 12 | 24 | Male | 17.301 | NA | NA | YES |
| 12×10^8^ particles | | | | | | |
| Volunteer 13 | 25 | Male | 22.567 | NA | NA | YES |
| Volunteer 14 | 29 | Male | 21.626 | NA | NA | YES |
| Volunteer 15 | 24 | Female | 19.948 | NA | NA | NO |
| Volunteer 16 | 26 | Male | 24.535 | NA | NA | NO |
| Volunteer 17 | 25 | Female | 18.590 | NA | NA | NO |
| Volunteer 18 | 25 | Female | 21.218 | NA | NA | NO |
| 16×10^8^ particles | | | | | | |
| Volunteer 19 | 26 | Male | 22.145 | NA | NA | NO |
| Volunteer 20 | 27 | Male | 23.451 | NA | NA | NO |
| Volunteer 21 | 25 | Female | 23.438 | NA | NA | NO |
| Volunteer 22 | 27 | Female | 20.761 | NA | NA | NO |
| Volunteer 23 | 24 | Female | 20.324 | NA | NA | NO |
| Volunteer 24 | 24 | Male | 23.875 | NA | NA | NO |

**Supplementary Table 2. Laboratory parameters before and after haMSCs-EVs nebulization in MEXVT**

|  | 2×10^8^ particles | | 4×10^8^ particles | | 8×10^8^ particles | | 12×10^8^ particles | | 16×10^8^ particles | |
| --- | --- | --- | --- | --- | --- | --- | --- | --- | --- | --- |
|  | Day 0 | Day 7 | Day 0 | Day 7 | Day 0 | Day 7 | Day 0 | Day 7 | Day 0 | Day 7 |
| WBC (×10^9^/L) | 6.99  (6.62-7.83) | 5.6  (5.1-8.6) | 6.73  (6.33-7.56) | 6.9  (6.63-7.5) | 5.6  (5.29-6.74) | 5.6  (5-6.75) | 5.695  (5.14-6.593) | 5.305  (4.958-6.395) | 5.92  (4.65-7.555) | 5.44  (4.955-6.135) |
| N (×10^9^/L) | 4.39  (4.19-4.42) | 3.3  (2.9-5.8) | 3.99  (3.55-4.84) | 3.55  (2.84-3.61) | 3.63  (2.79-4.23) | 2.885  (2.205-4.36) | 3.05  (2.79-3.473) | 2.465  (2.27-3.493) | 3.185  (2.3-4.068) | 3.36  (2.363-4.063) |
| LYM (×10^9^/L) | 2.1  (1.93-2.73) | 1.8  (1.6-2.2) | 2.13  (2.12-2.40) | 3.03  (2.62-3.13) | 1.76  (1.41-2.37) | 2.07  (1.583-2.29) | 2.045  (1.918-2.353) | 2.155  (2.043-2.71) | 2.07  (1.905-2.298) | 1.735  (1.575-1.885) |
| RBC (×10^12^/L) | 4.54  (4.22-4.58) | 4.27  (4.22-4.46) | 5.15  (4.37-5.24) | 5.37  (4.44-5.64) | 4.62  (4.47-5.6) | 4.795  (4.168-5.758) | 4.765  (4.103-5.288) | 4.56  (4-4.978) | 4.895  (4.638-4.975) | 4.555  (4.133-4.75) |
| Hb  (g/L) | 140  (135-148) | 135  (133-143) | 154  (132-154) | 155  (133-165) | 145.5  (141.5-148.3) | 148.5 (129.8-154.3) | 140.5  (124.3-153.3) | 133  (120-148) | 146.5  (135-153) | 133  (120-146.5) |
| PLT (×10^9^/L) | 224  (198-275) | 210  (205-274) | 270  (202-287) | 291  (201-297) | 249.5  (204.8-278) | 284.5  (193.9-316.5) | 224  (177.5-254) | 225.5  (167.8-246.8) | 236  (196-266.3) | 206.5  (174-221.8) |
| ALT (IU/L) | 16  (16-24) | 20  (16-21) | 21  (8-30) | 24  (9-24) | 24  (14.25-42) | 23.5  (13.25-58.25) | 17.5  (9.75-21.25) | 16  (11.75-23.75) | 15.5  (13.25-18.5) | 15.5  (11-18) |
| AST (IU/L) | 20  (20-22) | 18  (17-21) | 24  (18-26) | 21  (16-26) | 22  (19.75-26) | 24.5  (16.75-29.75) | 17  (15.75-22) | 19  (17.75-24) | 17  (15.75-21.5) | 16.5  (15-19.75) |
| ALP (IU/L) | 82  (64-92) | 85  (56-86) | 70  (40-81) | 74  (39-80) | 65  (54.25-76.25) | 68.5  (52.25-90.25) | 67  (43.5-78.75) | 66  (44.25-77) | 63  (53-72.75) | 59.5  (47.5-68.25) |
| TBil (μmol/L) | 8.9  (5.4-16.2) | 10.8  (10.4-30.4) | 12.9  (9.8-20.4) | 22.3  (16.2-22.6) | 11.55  (8.325-16.4) | 17.45  (11.68-22.9) | 12.15  (8.475-15.45) | 10.35  (8.675-15.68) | 13.7  (12-15.68) | 10.95  (8.6-16.7) |
| TP  (g/L) | 76  (74-76) | 71  (71-73) | 73  (71-78) | 76  (73-76) | 75  (71.25-78) | 73  (69-79) | 74  (71.5-77.25) | 69  (67-71.25) | 74  (73.75-77.5) | 68.5  (64.75-73.25) |
| Alb  (g/L) | 45  (45-62) | 43  (43-51) | 42  (42-48) | 46  (43-51) | 45.5  (44.5-47.25) | 45  (42.75-47.5) | 48.5  (46.25-49.5) | 44  (42-46.25) | 49.5  (48-51) | 44.5  (42.5-48) |
| BUN (mmol/L) | 4.6  (4.1-6.1) | 3.8  (3.1-5.2) | 3.8  (2.6-5.5) | 5.2  (3.9-5.2) | 4.8  (3.65-6.625) | 4.5  (3.4-5.75) | 4.15  (3.5-5.4) | 4.15  (3.525-4.6) | 4.25  (3.95-4.8) | 4.8  (4.125-5.175) |
| Cr (μmol/L) | 67  (64-86) | 64  (59-86) | 83  (57-106) | 77  (67-115) | 82.5  (62.75-90) | 86  (63.25-92.75) | 74.5  (59-95) | 74.5  (64.75-86.25) | 81  (56-91) | 80  (56.5-90) |
| GLU (mmol/L) | 5.42  (5.03-5.71) | 5.22  (4.93-5.4) | 5.09  (5.02-6.58) | 4.8  (4.3-5.01) | 5.89  (5.058-6.843) | 4.845  (4.608-5.015) | 4.825  (4.658-4.963) | 4.635  (4.52-4.798) | 4.82  (4.515-5.015) | 4.67  (4.463-4.763) |
| TG (mmol/L) | 1.4  (1.06-3.11) | 0.97  (0.8-1.03) | 1.21  (0.85-1.69) | 0.74  (0.67-0.91) | 1.47  (1.085-2) | 1.36  (0.755-1.743) | 1.405(0.6275-1.693) | 1.2  (0.62-1.473) | 0.835  (0.635-1.26) | 0.71  (0.5875-0.8025) |
| TC (mmol/L) | 4.69  (4.66-5.09) | 4.15  (3.85-5.17) | 4.71  (4.14-5.10) | 4.92  (4.4-5.37) | 4.37  (3.605-5.438) | 4.25  (3.858-5.195) | 4.805  (4.263-5.28) | 4.355  (3.593-4.718) | 3.84  (3.53-4.908) | 3.425  (3.105-4.77) |
| HDL (mmol/L) | 1.76  (1.38-1.92) | 1.77  (1.23-1.82) | 1.48  (1.42-1.6) | 1.55  (1.45-1.59) | 1.385  (1.02-1.703) | 1.385  (1.09-1.563) | 1.475  (1.113-1.9) | 1.395  (1.043-1.71) | 1.455  (1.28-1.585) | 1.325  (1.2-1.493) |
| LDL (mmol/L) | 2.61  (2.21-2.89) | 2.63  (1.9-2.96) | 2.85  (2.31-3.26) | 3.06  (2.68-3.63) | 2.465  (1.843-2.948) | 2.39  (2.11-2.915) | 2.7  (2.445-3.108) | 2.24  (2.1-3.018) | 2.165  (1.928-3.04) | 1.895  (1.658-3.055) |
| IgG  (g/L) | 11.75(10.46-13.02) | 10.99  (9.98-11.77) | 12.19  (10.83-12.93) | 12.6  (11.61-13.69) | 12.69  (10.57-14.97) | 13.13  (10.26-15.53) | 10.99  (9.698-13.1) | 10.48  (8.795-12.22) | 12.95  (11.36-13.48) | 12.32  (10-12.96) |
| IgA  (g/L) | 2.8  (2.1-3.36) | 2.77  (2.05-3.18) | 2.62  (2.22-3.54) | 2.82  (2.36-3.52) | 2.4  (1.633-3.255) | 2.385  (1.755-3.03) | 1.9  (1.688-2.618) | 1.725  (1.588-2.37) | 2.035  (1.673-2.958) | 1.88  (1.618-2.673) |
| IgM  (g/L) | 1  (1-3.68) | 0.97  (0.93-3.82) | 1.06  (0.5-1.32) | 1.15  (0.56-1.35) | 1.07 (0.9925-1.825) | 1.145(0.9575-1.795) | 1.27  (0.86-1.77) | 1.175  (0.77-1.515) | 1.125  (0.895-1.515) | 1.005  (0.8525-1.425) |
| IgE (IU/mL) | 13.2  (12.8-79.1) | 13.2  (9.4-79.2) | 65.6  (32.1-157) | 64.3  (28.4-143) | 13.2  (6.55-171) | 68.4  (8.35-144.5) | 144  (92.43-245) | 137  (83.3-226.8) | 20.65  (13.68-47.95) | 17.85  (12.65-49.1) |
| C3  (g/L) | 1  (0.9-1.25) | 1.04  (0.89-1.14) | 1.08  (0.9-1.21) | 1.12  (1.03-1.3) | 1.09  (0.995-1.118) | 1.105  (1.04-1.113) | 1.045  (1.01-1.075) | 0.985  (0.94-1.045) | 1.025  (0.995-1.093) | 0.955  (0.8875-1.003) |
| C4  (g/L) | 0.25  (0.19-0.34) | 0.27  (0.19-0.32) | 0.30  (0.24-0.42) | 0.33  (0.24-0.45) | 0.30  (0.2125-0.42) | 0.335  (0.19-0.485) | 0.22 (0.1375-0.265) | 0.21  (0.16-0.245) | 0.25  (0.1925-0.28) | 0.22  (0.1775-0.2625) |
| LDH (IU/L) | 172  (140-178) | 135  (109-143) | 193  (181-194) | 172  (165-174) | 153  (139.3-180.5) | 160.5  (138.5-166.8) | 157.5  (141.3-176.8) | 159.5  (151.5-178) | 170  (161.8-192.3) | 160.5  (144.5-194.8) |

**Data were shown in median with interquartile range (IQR).**

WBC: white blood cell; N: neutrophils; LYM: lymphocyte; RBC: red blood cell; Hb, hemoglobin; PLT: platelet; ALT: alanine aminotransferase; AST: aspartate aminotransferase; ALP: alkaline phosphatase; TBil: total bilirubin; TP: total protein; Alb: albumin; BUN: urea; Cr: creatinine; GLU: glucose; TG: triglyceride; TC: cholesterol; HDL: High-density lipoprotein; LDL: low-density lipoprotein; IgG: immunoglobulinG; IgA: immunoglobulinA; IgM, immunoglobulinM; IgE: immunoglobulinE; C3: Complement 3; C4: Complement 4; LDH: lactate dehydrogenase
